# Squeeze-Excitation Attention-Guided 3D Inception ResNet for Aflatoxin B1 Classification in Almonds Using Hyperspectral Imaging

**DOI:** 10.3390/toxins18020076

**Published:** 2026-02-02

**Authors:** Md. Ahasan Kabir, Ivan Lee, Sang-Heon Lee

**Affiliations:** 1 UniSA STEM, University of South Australia, Mawson Lakes, SA 5095, Australia; ivan.lee@unisa.edu.au (I.L.); sang-heon.lee@unisa.edu.au (S.-H.L.); 2 Department of Electronics and Telecommunication Engineering, Chittagong University of Engineering and Technology, Chittagong 4349, Bangladesh

**Keywords:** squeeze-excitation attention, aflatoxin B1, hyperspectral imaging, inception ResNet, attention guided deep neural network, attention mechanism

## Abstract

Almonds are a highly valued nut due to their rich protein and nutritional content. However, they are vulnerable to aflatoxin B1 (AFB1) contamination in warm and humid environments. Consumption of AFB1-contaminated almonds can pose serious health risks, including kidney damage, and may lead to significant economic losses. Consequently, a rapid and non-destructive detection method is essential to ensure food safety by identifying and removing contaminated almonds from the supply chain. Hyperspectral imaging (HSI) and 3D deep learning provide a non-destructive, efficient alternative to current AFB1 detection methods. This study presents an attention-guided Inception ResNet 3D Network (AGIR-3DNet) for fast and precise detection of AFB1 contamination in almonds utilizing HSI. The proposed model integrates multi-scale feature extraction, residual learning, and attention mechanisms to enhance spatial-spectral feature representation, enabling more precise classification. The proposed 3D model was rigorously tested, and its performance was compared against 3D Inception and various conventional machine learning models. Compared to conventional machine learning models and deep learning architectures, AGIR-3DNet outperformed and achieved superior validation accuracy of 93.30%, an F1-score (harmonic mean of precision and recall) of 0.94, and an area under the receiver operating characteristic curve (AUC) value of 0.98. Furthermore, the model enhances processing efficiency, making it faster and more suitable for real-time industrial applications.

## 1. Introduction

Aflatoxin B1 (AFB1) is a highly potent toxin and carcinogen, primarily produced by the fungi *Aspergillus flavus* and *A. parasiticus*. These fungi flourish in warm, humid conditions, increasing the susceptibility of foods like cereals, dried fruits, and nuts to contamination [1]. Almonds, due to their high nutritional content, are particularly susceptible to fungal colonization under suboptimal pre-harvest, post-harvest storage, or processing conditions. The impact of climate variability, such as intensified temperature and humidity fluctuations, further exacerbates the proliferation of these fungi, increasing the prevalence of AFB1 contamination in agricultural products [2,3]. Additionally, improper drying techniques and prolonged stockpiling further increase the risk of almond contamination [4,5].

AFB1 is a major global public health concern due to its severe health consequences. Prolonged dietary exposure to AFB1 is a significant risk factor for liver cancer, specifically hepatocellular carcinoma, which contributes to a substantial proportion of global cancer mortality [6]. Beyond cancer risks, AFB1 has been implicated in immune suppression, making individuals more susceptible to opportunistic infections, while also stunting growth and cognitive development in children. These health impacts are disproportionately felt in regions with limited access to food safety technologies [7,8]. Besides that, AFB1 contamination presents substantial challenges to global food industries. Almonds, a high-value export commodity, are particularly affected by strict regulatory standards enforced by international agencies such as the European Food Safety Authority and the United States Food and Drug Administration, which impose stringent limits on allowable aflatoxin levels in food products: 8 ppb and 20 ppb, respectively [9,10]. Failure to meet these standards can result in costly shipment rejections, product recalls, and reputational damage, significantly impacting both large-scale producers and smallholder farmers [11]. Additionally, the downstream costs of managing public health outcomes, including medical treatment for aflatoxin-related illnesses and loss of economic productivity, further highlight the broader societal burden of AFB1 contamination [9,12].

Given the interconnected health, economic, and regulatory challenges, implementing effective strategies for detecting and mitigating AFB1 is imperative. Therefore, almond industries require robust, non-invasive, and scalable detection methods to ensure food safety while maintaining economic sustainability. Traditional detection methods, such as high-performance liquid chromatography (HPLC) [13] and enzyme-linked immunosorbent assay (ELISA) [14], remain the gold standard due to their accuracy and sensitivity. However, these methods are inherently labor-intensive, destructive to samples, and not scalable for large-scale industrial use. The need for rapid, non-invasive, and efficient detection techniques has led to increased interest in optical and imaging-based technologies [2,3]. Hyperspectral imaging (HSI) has emerged as a leading technology in this domain, offering the potential to address these challenges by providing rapid, accurate, and non-destructive screening for aflatoxin contamination [15,16]. HSI captures information across a wide range of wavelengths, creating a comprehensive spectral profile for each pixel in an image. This spectral signature enables the differentiation of AFB1-contaminated almonds from uncontaminated ones by identifying subtle chemical variations associated with fungal metabolites.

The detection of AFB1 in cereals, grains, and nuts has attracted significant research attention due to its serious implications for public health and global food security. Zhou et al. [3] utilized near-infrared HSI to classify maize kernels contaminated with AFB1, achieving high accuracy by integrating feature selection methods. Similarly, Zhu et al. [9] applied short-wave infrared (SWIR) HSI coupled with deep learning to detect AFB1 contamination in peanut kernels. Their approach successfully mined critical wavelengths, enhancing the efficiency of spectral data processing. Bertani et al. [4] explored multispectral fluorescence imaging to detect AFB1 in ground peanuts, successfully identifying fluorescence markers associated with contamination. Wu et al. [2] demonstrated the efficacy of HSI in detecting aflatoxin-producing fungi in various nuts, integrating chemometric models for accurate quantification.

Research on the detection of AFB1 contamination in almonds has intensified due to their high economic value and significant health risks associated with AFB1 exposure. HSI has been particularly successful in this field, where traditional methods are often impractical. Torres-Rodríguez et al. [15] demonstrated HSI’s ability to differentiate bitter almonds from sweet ones based on spectral differences, while Ariza Ramirez et al. [17] employed SWIR HSI to detect AFB1 in single almond kernels. The application of chemometric models in these studies highlights the potential for real-time, non-destructive analysis. Mishra et al. [5] employed multispectral imaging to classify AFB1-contaminated almonds by integrating chemometric techniques with support vector machine (SVM) and random forest (RF) algorithms. Kabir et al. [18] extended this work by combining RF and quadratic discriminant analysis (QDA) with hyperspectral data, achieving high classification accuracy.

Previous research on AFB1 detection in almonds using HSI has mainly aimed to establish the feasibility of the technique under controlled conditions. These studies typically use almonds with similar thickness and geometry to maintain consistent spectral responses. While these efforts demonstrate the potential of HSI, they overlook the inherent variability in real-world almond samples, where differences in morphology, surface texture, and thickness can significantly alter spectral signatures, leading to model inaccuracies. Furthermore, conventional machine learning models such as SVM, quadratic discriminant analysis (QDA), RF, and partial least squares regression (PLSR), which are commonly used in prior works, exhibit critical limitations. SVM and QDA primarily rely on handcrafted feature representations and assume relatively simple decision boundaries, which limits their effectiveness in capturing complex nonlinear patterns. RF improves robustness through ensemble learning but remains dependent on predefined features and struggles with very high-dimensional inputs. PLSR is effective for dimensionality reduction and linear regression but is inherently limited in modeling nonlinear spectral-spatial interactions. As a result, these approaches face significant challenges when applied to 3D HSI data, which are characterized by high dimensionality, strong spectra-spatial coupling, and nonlinear relationships. These limitations reduce their generalization capability across heterogeneous almond samples, thereby constraining their practical deployment in real industrial environments where sample variability is unavoidable [19,20].

Our previous deep learning study [21] addressed several of these modeling limitations by introducing deep 3D Inception and 3D Inception-ResNet architectures, achieving high classification accuracy for AFB1 detection. However, the most accurate models were computationally intensive, with very deep architectures and large parameter counts, making them unsuitable for real-time, inline industrial deployment. Although a lightweight variant improved efficiency, inference speed and computational scalability remained limiting factors for practical, high-throughput sorting systems. These limitations highlight the need for a computationally efficient, attention-aware 3D deep learning framework that preserves classification performance while enabling real-time industrial applicability.

To address these challenges, this study introduces a squeeze-and-excitation (SE) attention-guided Inception-ResNet 3D Network (AGIR-3DNet) architecture specifically designed for computationally efficient processing of raw HSI data in industrial settings. By integrating channel-wise attention within a compact inception–residual framework, the proposed model adaptively emphasizes contamination-relevant spectral–spatial features while suppressing redundant information, thereby improving efficiency and robustness. The lightweight design of AGIR-3DNet significantly reduces network depth, parameter count, and inference time compared with previously reported 3D Inception-ResNet models, while maintaining competitive classification performance. This architecture enables invariant feature learning across heterogeneous almond geometries and surface textures without reliance on extensive preprocessing or shape normalization, addressing a key limitation of earlier deep learning approaches. This research contributes (1) an attention-guided and deployment-oriented 3D deep learning architecture optimized for hyperspectral data, (2) empirical validation of efficiency-aware deep learning for robust spectral-spatial feature extraction under real-world variability, and (3) a practical and scalable pathway for real-time AFB1 monitoring in inline industrial quality control systems, supporting improved food safety and operational feasibility.

## 2. Results and Discussion

**Figure 1:**
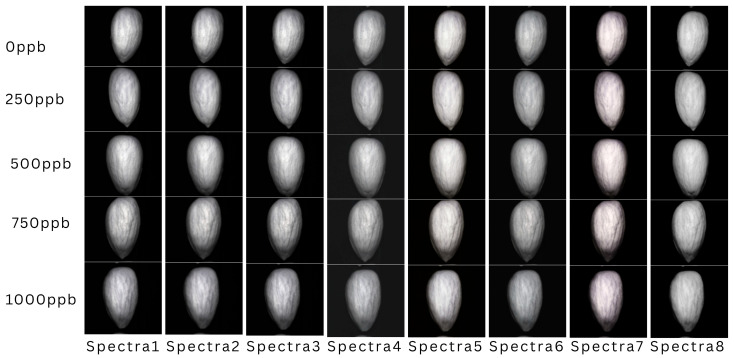
Representation of normal and contaminated almonds using 3 selected hyperspectral bands.

We proposed an AGIR-3DNet to classify AFB1 in almonds using multispectral images. The developed model was evaluated using an artificially contaminated AFB1 multispectral image dataset containing almonds with varying shapes, thicknesses, and textures. These variations affected the multispectral responses, making it challenging for the machine-learning model to achieve high classification accuracy. Figure 1 presents images of normal and contaminated almonds constructed from each selected spectral band (8 spectral bands). Despite this visualization, differentiating contaminated from non-contaminated almonds by eye remains extremely difficult. However, the proposed AGIR-3DNet model effectively detected AFB1-contaminated almonds using multispectral images, surpassing classical machine learning methods across all quantitative and qualitative metrics. In the experiment of this comparison study, all 3D deep learning models were trained with the Adam solver, L_2_ regularization, an initial learning rate of 0.001, a learning rate drop factor of 0.1, and a minibatch size of 64. The best network was selected based on the lowest validation loss. The model was developed on a class-balanced dataset of 3596 almond samples, which was split 50:25:25 for training, testing, and validation, respectively, to ensure the developed model performed well on the validation dataset. All machine learning experiments were conducted in MATLAB 2024b using a workstation with an Intel Xeon E5-2620 v3 processor and 32 GB of system memory. The CAES-HBS feature selection algorithm, as described in Section 4.3, was used to reduce the full hyperspectral image data to 4, 6, and 8 of the most significant spectral bands. Model performance was then evaluated to assess improvements as the number of selected spectral bands increased. For comparison, a trial using the full set of spectral bands was also conducted to demonstrate the accuracy and robustness of the proposed AGIR-3DNet model when applied to complete hyperspectral data. Several traditional machine learning models-linear SVM (box constraint = 10, standardization = on, kernel = linear) [22], QDA (regularization = 0.01, pseudo-) [23], AdaBoost (100 weak learners, learning rate = 0.1) [24], RUSBoost (100 weak learners, learning rate = 0.1, random under sampling) [25], and Subspace ensemble learning (100 learners) [26]—along with 3D Inception, Inception-ResNet (Deep), and Inception-ResNet (Light) models, were evaluated for performance comparison with the proposed model. The evaluation metrics included accuracy, F1-score, and Area Under the Curve (AUC) [27,28] for different spectral configurations. The F1-score is the harmonic mean of precision and recall, providing a balanced measure of a model’s performance on both positive and negative classes, while AUC quantifies the model’s ability to distinguish between classes across all classification thresholds, with higher values indicating better discriminative performance. For the experiment of the traditional ML models, a comprehensive feature extraction process was carried out, resulting in a total of 55 distinct features. These features were selected to capture complementary spatial, frequency, and texture characteristics relevant to contamination-induced spectral-spatial variations. Specifically, 16 texture features were computed from gray-level co-occurrence matrices, including contrast, correlation, energy, and homogeneity across multiple orientations. Four statistical features (mean, variance, skewness, and kurtosis) were extracted to describe the intensity distribution. Sixteen Gabor filter responses were obtained using four orientations and four scales to capture directional texture patterns. Two discrete cosine transform coefficients and two Fourier transform features were selected to represent dominant frequency-domain components. Eight discrete wavelet transform features were derived from multi-level wavelet decomposition, capturing both low- and high-frequency information. In addition, five gradient-based features were extracted to describe edge and intensity transition information, and two scale-invariant feature transform descriptors were included to capture local invariant key point characteristics. The combination of these features was designed to provide a comprehensive representation of spectral-spatial information for conventional machine-learning classification. The combination of these varied features could empower traditional machine learning algorithms to deliver an acceptable level of strong and reliable performance.

The validation metrics of all selected machine learning models are tabulated in Table 1. A 5-fold cross-validation was used to evaluate all classical machine learning models. Traditional machine learning models show moderate performance across different spectral configurations, with SVM and Subspace ensemble learning achieving the highest validation accuracy and F1 scores. For example, SVM reaches 78.13% accuracy and a 0.76 F1-score for 8 spectra but drops to 76.54% accuracy with full spectra, suggesting difficulty in leveraging high-dimensional information and the potential inclusion of irrelevant spectral bands. The full-spectrum model used 10 principal components (PCs) to reduce model complexity while explaining 99.99% of the data. Pseudo-QDA performs the worst, with accuracy ranging from 66.45% to 70.10%, likely due to its assumption of Gaussian-distributed data, which does not hold for hyperspectral almond images. AdaBoost and RUSBoost ensemble models perform more consistently, with AdaBoost achieving 75.05% accuracy and a 0.75 F1-score on full spectra, indicating that ensemble methods help mitigate some limitations of individual classifiers.

As Table 1 shows, 3D Inception models outperform traditional machine learning approaches in capturing spectral-spatial correlations, achieving 86.92% accuracy for 8 spectra, though performance declines with full spectra. The full-spectrum models generally show lower performance, as hyperspectral images include both AFB1-sensitive and irrelevant bands. PCA reduces dimensionality but treats all information equally, which can dilute relevant signals. In contrast, the feature selection method identifies and retains only the most informative spectral bands, improving classification performance. These results highlight that model architecture, spectral selection, and feature representation all significantly influence the effectiveness of AFB1 detection in almonds, providing guidance for future hyperspectral imaging studies.

The proposed AGIR-3DNet consistently outperforms all other models across spectral configurations, achieving superior results, including 93.30% accuracy, a 0.94 F1-score, and a 0.98 AUC for 8 spectra. The model demonstrates robustness, maintaining high accuracy across various spectral bands (87.03% for 4 spectra, 89.30% for 6 spectra, and 88.53% for full spectra). F1-scores remain consistently high (0.86–0.94), and AUC values are close to 1.0, indicating strong discriminative power. This superior performance is driven by the use of multiscale feature extraction, multi-scale Inception ResNet modules, squeeze-and-excitation blocks for feature refinement, and an efficient 3D architecture that reduces computational complexity while maintaining high performance. The performance of all models generally improves as the number of spectral bands increases from 4 to 8 but slightly degrades when using the full spectra. The proposed model experiences minimal degradation, with only a 5.11% drop in accuracy from 8 to full spectra, compared to a 9.46% drop for the 3D Inception model, showcasing its robustness to high-dimensional data. Achieving 93.30% accuracy with just 8 spectral bands demonstrates the model’s potential for real-world aflatoxin detection applications, offering reduced computational costs and faster data acquisition while maintaining high performance, making it ideal for industrial deployment. It is important to note that the accuracy improvement over the deep 3D Inception-ResNet baseline is modest, confirming that the proposed model does not introduce a fundamentally new detection capability. Instead, AGIR-3DNet achieves comparable or slightly improved classification performance with substantially lower computational complexity, making efficiency, not accuracy, the principal contribution of this study.

Table 2 presents the performance metrics for two variants of the AGIR-3DNet architecture: with SE blocks and without them, evaluated across three network sizes. Here, ‘AGIR’ stands for the attention-guided Inception ResNet block. In all three network sizes, the number of multiscale feature extraction and AGIR blocks varies, while the stem block remains the same. The performance of both variants improves significantly as the network size increases, AGIR from 1× to 2× and 3×. The 1× network performed poorly because the network was too shallow to effectively learn the complex spatial-spectral features in the hyperspectral data. In contrast, the 2× network shows a dramatic improvement, achieving testing and validation accuracies exceeding 93% for the variant with SE blocks. However, the 3× network exhibits slightly reduced performance compared to the 2× network with SE, suggesting diminishing returns and potential overfitting risks in larger models. The inclusion of SE blocks enhanced performance across all network sizes, as evidenced by higher accuracy, F1 scores, and AUC values. In the 2× network, validation accuracy improves from 90.60 (without SE) to 93.30% (with SE), testing accuracy from 89.11 to 93.92%, and AUC rises from 0.96 to 0.98, demonstrating their effectiveness in feature recalibration and improving generalization. The experimental result shows that the 2× network with SE blocks achieves an optimal balance between complexity and performance, surpassing 93.3% testing accuracy. These results confirm that the optimal 2× AGIR-3DNet with SE blocks delivers the best trade-off between accuracy and efficiency, validating its suitability for inline, high-throughput industrial deployment rather than purely performance-driven benchmarking.

We also compared the number of floating-point operations (FLOPs) [29] and parameters across different variants of AGIR-3DNet models, both with and without the SE module. The complexity of the model increases as the depth (number of AGIR layers) increases. For the AGIR-3DNet with the SE variant, the FLOPs and parameters rise from 44.7 G and 10 M for the 1× AGIR model to 132.6 G and 25.6 M for the 3× AGIR model. Similarly, the AGIR-3DNet without SE exhibits a lower computational cost, with FLOPs increasing from 40.27 G to 119.4 G and parameters from 6.9 M to 19.4 M as the depth increases. The SE module contributes to a slight increase in FLOPs and parameter count, indicating a trade-off between computational complexity and potential performance improvements. SE blocks play a crucial role in improving generalization and robustness, enhancing the model’s effectiveness. The results highlight the superiority of AGIR-3DNet with SE blocks for hyperspectral image classification tasks.

The computational efficiency of different model architectures was evaluated based on the time required to classify a fixed test set of 899 samples. The proposed AGIR-3DNet model variants demonstrated significantly faster testing performance. For the AGIR-3DNet architecture without the SE attention module, the testing times were 2.18, 2.34, and 2.36 s for the 1×, 2×, and 3× AGIR configurations, respectively. When the SE module was removed, the testing times slightly increased to 2.41, 2.43, and 3.75 s for the respective AGIR model depths. This may be because SE blocks suppress less useful features and amplify important ones, resulting in more focused and cleaner feature maps [30], which reduce internal model complexity and speed up forward propagation. These results highlight that even the deepest version of AGIR-3DNet with attention remains computationally efficient, significantly outperforming proposed 3D deep learning models in testing time. The previously developed 3D deep learning models, the 3D Inception-ResNet at 7.49 s, while the 3D Inception model demonstrated improved efficiency with a testing time of 4.76 s. The comparison of computational costs clearly demonstrates the proposed model’s suitability for real inline industrial applications, highlighting its efficiency and scalability across different network configurations while maintaining competitive performance relative to traditional machine learning and deep learning models.

While this study demonstrates strong classification performance within the evaluated AFB1 concentration range, determining the precise limit of detection (LOD) would require further validation at lower contamination levels. Such validation would involve finer concentration intervals, larger sample sizes at near-threshold levels, and potentially regression-based modeling approaches. Future work will explore integrating HSI with targeted chemical quantification methods to establish a robust LOD. From an industrial perspective, the proposed system is designed for rapid, non-destructive screening and early risk identification rather than precise toxin quantification.

## 3. Conclusions

In this study, we developed an AGIR-3DNet to address the critical challenge of detecting AFB1 contamination in almonds utilizing HSI. By integrating multiscale feature extraction, Inception residual connections, and SE blocks, the proposed architecture demonstrated superior performance compared to existing methods, achieving an accuracy of 93.30%, an F1 score of 0.94, and an AUC value of 0.98. These results underscore the model’s ability to capture intricate spatial-spectral correlations inherent in hyperspectral data, enabling precise identification of contaminated almonds, a significant advancement for food safety applications. The success of the architecture stems from its synergistic components: multiscale feature extraction blocks extract low to medium-scale features between spatial regions and spectral bands, while SE blocks dynamically prioritize discriminative wavelengths linked to AFB1’s molecular signatures. Residual connections ensured stable training, mitigating gradient degradation even in deeper layers. Ablation studies and statistical validation confirmed the importance of each design choice, with SE blocks enhancing validation and testing accuracy by around 3% for the optimal length network (2×). This model has the potential to be used in an inline real-time industrial application. Unlike prior studies that prioritized maximum classification accuracy, this work demonstrates that comparable detection performance can be achieved with significantly reduced computational complexity, positioning AGIR-3DNet as a deployment-ready solution rather than a purely performance-oriented model.

Despite these advancements, the dataset’s restriction to a single almond cultivar, high contamination level, and limited sample size may constrain generalizability to diverse agricultural conditions or other toxins. Future efforts should expand datasets to include varied cultivars with lower contamination. However, by combining cutting-edge deep learning with HSI, the proposed model establishes a foundation for automated, real-time detection of AFB1 contamination in almonds, offering promising applications in industrial sorting systems and ensuring adherence to regulatory standards.

## 4. Materials and Database Preparation

The hyperspectral dataset used in this study is intentionally the same as that employed in our previous work. This choice enables a fair architectural comparison by isolating the effects of network design, attention mechanisms, and computational efficiency from data-related variability. Accordingly, this study does not introduce new data or samples but focuses on evaluating the deployment-oriented performance of the proposed AGIR-3DNet architecture.

### 4.1. Almond Sample Selection and Preparation

Almonds of the Nonpareil variety were selected for this study due to their commercial significance in Australia. The samples were sourced from a processing plant located in Renmark, South Australia, during the 2022–2023 season. To maintain quality and prevent degradation, the collected almonds were stored under refrigerated conditions (4 °C) until further analysis. Since it is not possible to identify naturally contaminated almonds in a non-destructive manner, the samples were artificially contaminated with AFB1 in a controlled environment [5,31,32]. To achieve contamination, 5 mg of standard *Aspergillus*-produced AFB1 was procured from Sigma Aldrich (Merck Life Science Pty Ltd., Bayswater, VIC, Australia). This was dissolved in 5 mL of a methanol-water solution (50:50) to prepare a 1 mg/mL stock solution, which was then further diluted with the same solvent mixture to create standard solutions of various contamination levels of 0.25, 0.5, 0.75, and 1.00 µg/g. Then, contamination-free almond kernels were individually inoculated with 20 µL of the prepared solutions, ensuring contamination levels of 250, 500, 750, and 1000 ppb, respectively. For analytical consistency, each almond was standardized to 1 g in weight. It is important to note that higher concentrations of AFB1 were intentionally used because previous studies show that naturally contaminated almonds rarely exceed 0.03% contamination [33]. This ensures that higher contamination has a large impact on AFB1 test results while allowing for efficient spectral detection, which is essential for industrial inline applications. After inoculation, the almonds were air-dried in a fume hood for 48 h, allowing complete evaporation of the methanol-water solution. A set of contamination-free control almonds (with 0 ppb AFB1) was also included as untreated samples. In total, 5400 almond kernels were prepared, comprising both contaminated and control samples, and stored under appropriate conditions for HSI and chemical analysis.

### 4.2. Hyperspectral Image Acquisition

Hyperspectral images of the prepared almond samples were captured using the camera system consisting of a SWIR InGaAs camera (Oulu, Finland), a lighting source, a conveyor belt, and a computer with Lumo scanner software (2019). The FX17e hyperspectral camera is designed for high-speed spectral imaging, capturing 527 frames per second across its full spectral range of 900–1700 nm with an 8 nm mean full width at half maximum (FWHM) spectral resolution. It is thermoelectrically cooled to enhance sensitivity and stability, delivering a 640-pixel spatial resolution and 1000:1 signal-to-noise ratio (SNR). The camera has 99.5% pixel operability, ensuring high-fidelity spectral data acquisition. Also, the calibration of the system was performed using white and dark reference images to correct for lighting inconsistencies and background noise. The FX17e is highly adaptable, offering 224 customizable spectral bands. The field of view under the camera was illuminated using Halogen lamps. Each almond kernel was placed individually on a conveyor belt that moved at a calibrated speed of 15 mm/s, ensuring consistency in sharp and accurate image acquisition. Each hyperspectral image was captured by scanning 200 lines, with each line containing 640 pixels, resulting in a spatial resolution of 200×640 pixels per spectral band. Given that the camera operates with 224 spectral bands, the final hyperspectral image had a dimensionality of 200×640×224, encompassing both spatial and spectral information.

### 4.3. HPLC Analysis for Sample Reference

To validate the contamination procedure and AFB1 contamination levels used in this experiment, a separate chemical analysis using HPLC was also conducted. The procedure of chemical analysis using HPLC is as follows. Eight contaminated almonds (to fit in a vial tube) were ground homogeneously, and 40 mL of methanol-water (1:1) was added before filtration through 0.45 µm filters. The mixture was shaken using a shaker for 1 h to ensure AFB1 dissolved in methanol and water. The aliquots were rested to settle down, and the slurry was filtered at least 1 mL for the HPLC test. The extracted aliquots were injected into an Agilent 1200 HPLC system (Santa Clara, CA, USA) equipped with a UV light source and a fluorescence detector for precise AFB1 analysis. A reverse-phase column was employed, operating with an excitation wavelength of 365 nm and a detection wavelength of 440 nm, under a pressure range of 140–160 kg/cm^2^. The mobile phase consisted of water, acetonitrile, and methanol in a 1:1:3 ratio, flowing at 1 mL/min. Eight samples were prepared for HPLC testing for each category (0, 250, 500, 750, 1000 ppb). Some representative HPLC chromatograms (Figures S1–S5) for different aflatoxin B1 concen-trations are provided in the Supplementary Materials section. In addition to in-lab testing using HPLC, the prepared samples were sent to an external certified laboratory to further validate the experimental procedure. An additional three sets of 20 almonds were sent to the National Measurement Institute (NMI), Australia, for independent validation. The comparative results from both tests confirmed the consistency of the contamination process, allowing the assigned contamination levels to be used as reference values for developing AFB1 detection models. In the measurement process, standard calibration curves were developed with a regression coefficient (R^2^) value of 0.99 using certified AFB1 solutions at known concentrations. After the consistency in the AFB1 contamination procedure was validated, the artificially contaminated concentration was considered ground truth. HPLC was employed to confirm the presence and consistency of AFB1 contamination in prepared samples as a quality control measure. The reference contamination levels used in this study were determined based on controlled spiking concentrations during sample preparation and were used as ground truth labels for model training and evaluation. HPLC-derived quantitative values were not used for calibration or validation of the proposed models, as the focus of this work is on non-destructive screening and classification rather than chemical quantification.

### 4.4. Hyperspectral Image Segmentation

In a hyperspectral camera, dark current noise arises even when the camera shutter is closed, and since it is temperature-dependent, it can introduce unwanted noise into the acquired images. To minimize this effect, the hyperspectral images were normalized using a standard white reflectance reference for spectral intensity correction. The white reflector reflects 99.9% of its incident light. This process required capturing a dark current image ($I_{dark}$) and a white reference image ($I_{white}$) immediately after acquiring the raw image ($I_{raw}$). The normalization process followed Equation (1) [5]:

(1) $$I=\left(I_{raw}-I_{dark}\right)/\left(I_{white}-I_{dark}\right)$$

After spectral normalization, the almond samples were segmented from the background of the acquired images to ensure the background would not affect deep learning model development. A pseudo-colored image was generated by considering the spectra 1176 nm as red, 1173 nm as green, and 1156 nm as blue, respectively. This image was converted into the hue, saturation, and value (HSV) color model. A threshold was applied to the V channel to identify the region of interest (RoI) pixels of the almond, which were then used to create a binary mask for segmentation. The segmentation was applied to all acquired images across the entire spectrum using the same binary mask. We acquired a total of 5400 images, and each image has a dimension 200×640×224. To align the segmented RoI with the input requirement of the deep learning architecture, all RoIs were resized to dimensions of 150×100×224 dimensions. Additionally, to correct for class imbalance in the dataset, a random subset of 1804 samples from the higher AFB1 contamination groups (500, 750, and 1000 ppb) was removed. This adjustment reduced the total dataset size to 3596 samples, consisting of 1798 samples for 0 ppb, 892 for 250 ppb, 706 for 500 ppb, and 100 samples each for the 750 ppb and 1000 ppb contamination levels.

### 4.5. Spectral Dimensionality Reduction

The prepared dataset contains 224 spectra, which is too many for real-time industrial applications. Therefore, selecting only the relevant and significant features is essential to minimize the number of feature spectra used in deep learning for real-time processing. To minimize the dimensionality of the complete hyperspectral dataset and streamline model complexity, principal component analysis (PCA) was utilized. PCA transforms high-dimensional spectral data into a lower-dimensional space by identifying new orthogonal axes, known as principal components (PCs), that capture the maximum variance in the data. This study used the first 10 PCs to develop a reduced-dimensionality version of the full spectral model. It is important to note that PCA was applied solely for comparison purposes between full-spectral and multispectral machine learning models.

For multispectral feature selection, a specialized algorithm, correlation-awareness evolutionary spectral hybrid band selection (CAES-HBS), was employed to identify key individual wavelengths that preserve class separability while reducing redundancy [34]. This distinction ensures that the multispectral models are based on physically meaningful and interpretable spectral bands. The CAES-HBS algorithm optimizes hyperspectral band selection for AFB1 detection in almonds by systematically reducing redundancy while maintaining classification accuracy. This algorithm uses the mean spectrum dataset and works in two main steps. First, the algorithm individually selects important spectra using various tree-based boosting ensemble methods and multilayer perceptron neural networks. It begins with preprocessing and normalization, followed by ranking spectral bands using boosting techniques such as AdaBoost, LogitBoost, and RUSBoost. A multilayer perceptron (MLP) further refines feature selection based on learned weights, while a modified genetic algorithm (GA) optimizes the selection by iteratively refining spectral subsets through crossover and mutation. Then, the algorithm refines the selected spectra through a correlation-aware evaluation, applying a sparse spectral band selection process to achieve optimal feature representation. Finally, it eliminates redundant bands, ensuring that only the most informative spectral features are retained. Using this approach, the most significant spectral bands were selected from the full set of 224 bands. To compare performance, the top 4, 6, and 8 most significant bands were evaluated, allowing an assessment of how the number of selected bands affects overall accuracy and efficiency.

## 5. Research Methodology

AGIR-3DNet is an architectural evolution motivated by the deployment constraints identified in our previous work, where deep 3D Inceptio-ResNet models achieved high classification accuracy but were limited by excessive depth, large parameter counts, and long inference times that restricted real-time industrial applicability. In contrast to the previously reported 3D Inception-ResNet architecture, which prioritized representational depth, AGIR-3DNet adopts a lightweight and efficiency-oriented design that explicitly targets fast inference and scalability for inline industrial sorting systems. A key distinction of AGIR-3DNet is the integration of SE attention modules-absent in earlier models—which enable adaptive channel-wise recalibration of spectral-spatial features, improving efficiency and robustness while reducing computational overhead.

**Figure 2:**
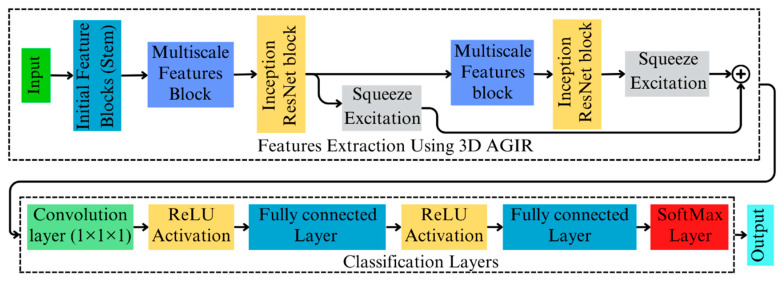
Framework of the proposed AGIR-3DNet architecture.

In this research study, we proposed a 3D deep neural network by integrating Inception ResNet features along with the SE attention mechanism. Figure 2 presents a brief schematic diagram of the AGIR-3DNet algorithm proposed in this study. The overall architecture is divided into two main components: feature extraction and classification. The AGIR-3DNet begins with an initial feature extraction module, referred to as the Stem, which is followed by a multiscale feature extraction block. These extracted features are then refined using an attention-guided Inception ResNet block to enhance relevant information before passing it to the classification block. The detailed structure and functionality of each component are explained in the following subsections.

### 5.1. AGIR-3DNet Architecture

The AGIR-3DNet is specifically designed to detect and classify AFB1 contamination in almonds using multispectral imaging. The design unifies the strengths of four key components: residual learning, multi-scale feature extraction, parameter reduction, and channel-wise recalibration (SE attention module). The architecture initiates with an initial feature block (Stem) that extracts fundamental low-level spectral-spatial patterns from the input multispectral images. This is followed by a multiscale features block, designed to capture information at multiple receptive fields through parallel convolutional pathways, addressing the varied sizes and distributions of contamination regions. The extracted features are then passed to an Inception-ResNet block, which combines the representational strength of Inception modules with the optimization benefits of residual learning, mitigating vanishing gradient problems in deeper networks. To further refine the feature maps, a SE attention module is integrated within this block, adaptively recalibrating channel-wise responses and ensuring emphasis on the most informative spectral spatial data. Then, the feature extraction process is enhanced by adding a sequence of multiscale feature blocks and Inception-ResNet blocks. Each Inception-ResNet block is paired with an SE attention module, enabling the network to progressively refine its learned representations.

After the feature extraction stage, the network transitions into a classification block, beginning with a 1 × 1 × 1 convolutional layer comprising 2080 filters, which compresses the multidimensional feature maps while preserving their salient characteristics. A ReLU activation is used to introduce non-linearity. The activated features are then processed by a fully connected layer with 2080 units, followed by another ReLU activation to maintain model expressiveness. Subsequently, a second fully connected layer with 2 output units projects the features onto class scores representing contaminated and non-contaminated classes. These class scores are finally processed by a SoftMax layer, converting them into probability estimates and yielding the model’s final prediction. The AGIR-3DNet integrates 3D convolutional operations, residual learning, and multiscale feature extraction to jointly model complex spatial-spectral dependencies in multispectral imaging data. By incorporating Inception-ResNet modules and SE attention modules, the network adaptively recalibrates feature responses to emphasize informative patterns while reducing noise and redundancy. Its lightweight, unified structure enhances classification accuracy and robustness to sample variability, offering a computationally efficient solution suitable for real-time AFB1 detection in industrial quality control environments. The detailed architecture and functionality of each component are explained in detail below.

### 5.2. Initial Feature Extraction Block

**Figure 3:**
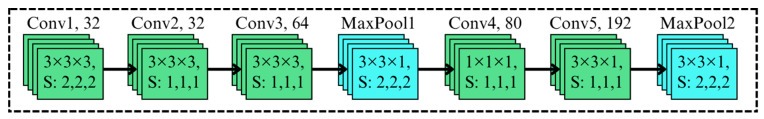
Illustration of initial feature extraction blocks (stem).

The multispectral image is fed to a 3D input layer, and initial features are extracted using a stem block, as shown in Figure 3. This block is designed to efficiently process input data while reducing spatial dimensions and increasing channel depth. It begins with three 3 × 3 × 3 convolutional layers followed by a 3 × 3 × 1 max pooling layer to capture large-scale features and downsample the input. Subsequently, two consecutive 1 × 1 × 1 and 3 × 3 × 1 convolutional layers were applied, further reducing spatial resolution while expanding the number of channels. Then, a 3 × 3 × 1 max pooling layer was used to downsample the input again. The Stem block ensures robust gradient flow and enables effective hierarchical feature extraction.

### 5.3. Multiscale Feature Extraction Block

**Figure 4:**
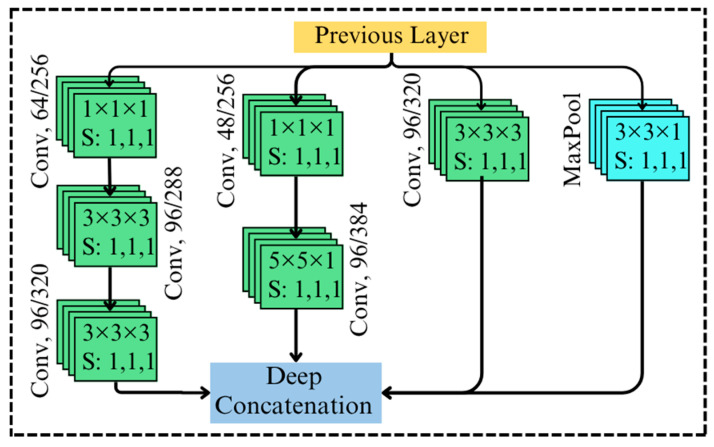
Multiscale feature extraction blocks of the proposed AGIR-3DNet.

The multiscale feature extraction block in the proposed AGIR-3DNet is designed to extract necessary features with various scales by leveraging parallel convolutional operations, as shown in Figure 4. This architecture enables the capture of fine-grained and coarse-grained spatial and spectral features, which are critical for high dimensional image data classification. The proposed 3D model employs volumetric convolutions in various steps, effectively handling the high-dimensional data. It extends 2D convolutions to three dimensions for processing volumetric data. The convolution of input, $I\in R^{H\times W\times D}$ (where H, W, and D represent the height, width, and depth of the input tensor) with a 3D convolutional kernel $W\in R^{m\times n\times p}$ (where m,n,p is the kernel dimension) can be expressed as [35]:

(2) $$x_{i,j,k}=\left(I*W\right)\left(i,j,k\right)=\sum_{m}\sum_{n}\sum_{p}x_{i-m,\;j-n,\;k-p}.W(m,n,p)$$

Batch normalization is applied after each 3D convolution layer to normalize the input features, stabilizing the learning process and accelerating convergence. The process ensures that the input to each layer has a consistent distribution, which mitigates the internal covariate. The input $x_{i,j,k}$ representing the feature at the spatial location i,j,k in channel c, is normalized using the mean $\left(\mu_{c}\right)$, small constant ϵ and variance $\left(\sigma_{c}^{2}\right)$ computed over the mini-batch [36]:

(3) $${\hat{x}}_{i,j,k}=x_{i,j,k}-\mu_{c}/\sqrt{\sigma_{c}^{2}+\epsilon }$$

After normalization, a learnable affine transformation is applied to ensure that the network retains its capacity to represent complex features, so the batch normalization output $y_{i,j,k}$ as:

(4) $$y_{i,j,k}=\gamma_{c}{\hat{x}}_{i,j,k}+\beta_{c}$$

here, $\gamma_{c}$ (scale) and $\beta_{c}$ (shift) are learnable parameters that allow the network to adaptively adjust the normalized features to the desired scale and offset. Also, strides of [1, 1, 1] and ‘same’ padding were employed to ensure the same output dimensions of each parallel branch.

The output of the 3D multiscale feature extraction block is a concatenation of feature maps derived from multiple branches operating at different spatial and spectral scales. Mathematically, if the output y is expressed as:

(5) $$y=\left[F_{1}\left(I\right),F_{3}\left(I\right),F_{5}\left(I\right),F_{pool}\left(I\right)\right]$$

where $F_{1}\left(I\right)$, $F_{3}\left(I\right)$ and $F_{5}\left(I\right)$ represent 3D convolution operations. Specifically, $F_{1}\left(I\right)$ uses 1×1×1 and 3×3×3 kernels; $F_{3}\left(I\right)$ uses 1×1×1 and 5×5×1 kernels; and $F_{5}\left(I\right)$ applies a 3×3×3 kernel. Additionally, $F_{pool}\left(I\right)$ denotes a 3D max pooling operation. Note that, in the first stage, a relatively lower number of filters were used, while a higher number of filters were applied in later stages. 1×1×1 convolution layer extracts fine-grained spectral features, the 3×3×1, 3×3×3 convolution captures mid-level spatial and spectral patterns, and the 5×5×1 convolution identifies broader contextual information. The pooling branch summarizes global features while reducing dimensionality, ensuring compact representations.

### 5.4. Attention-Guided Inception ResNet Block

The attention-guided Inception-ResNet framework, enhanced with squeeze-and-excitation attention, refines feature selection by dynamically recalibrating channel importance. This integration strengthens the ability of the network to capture diverse spatial and spectral patterns, which is particularly beneficial for hyperspectral image classification.

#### 5.4.1. 3D Inception ResNet

**Figure 5:**
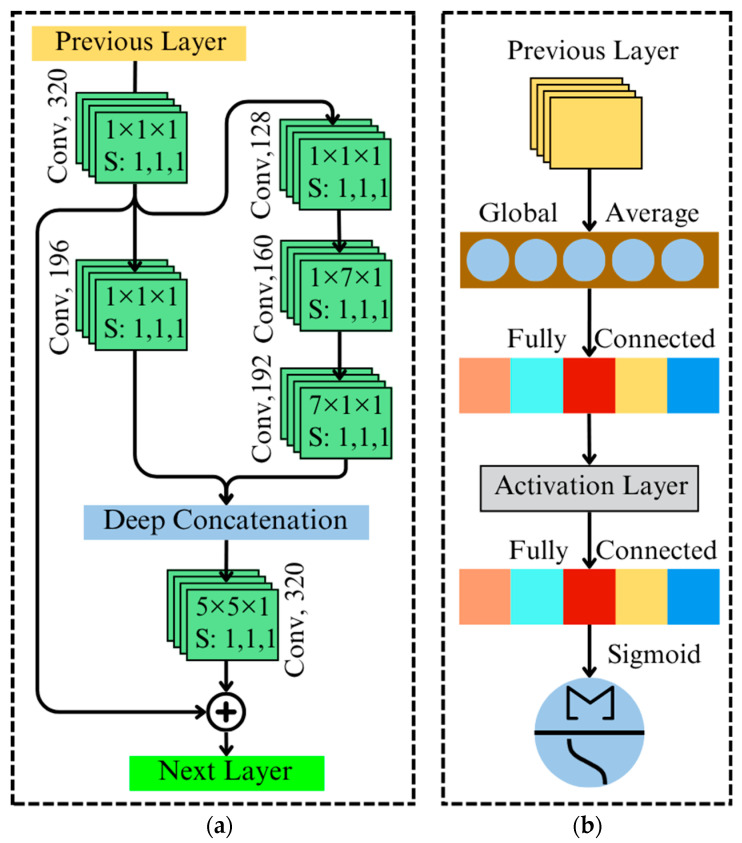
Illustration of (**a**) Inception ResNet and (**b**) squeeze-and-excitation attention block of the proposed AGIR-3DNet.

The Inception ResNet block introduces residual learning with multi-scale feature extraction, which alleviates the vanishing gradient problem in deep neural networks by enabling gradient flow through shortcut connections [21,37]. This block consists of three paths with different filter kernel sizes 1×1×1, 1×7×1, and 7×1×1 as illustrated in Figure 5a. Using 1×7×1 and 7×1×1 kernels in consecutive convolutional layers enable anisotropic feature extraction, where one layer captures spatial patterns while the other focuses on spectral correlations. This approach enhances feature discrimination in HSI while reducing computational complexity compared to standard isotropic 3D convolutions. For an input tensor $\bar{I}$, the residual output y is [37]:

(6) $$y=F\left(\bar{I},\left\{W_{i}\right\}\right)+\bar{I}$$

where $F\left(\bar{I},\left\{W_{i}\right\}\right)$ represents the multiscale residual mapping, which includes convolution, batch normalization, and activation operations. while $W_{i}$ is the learnable weight parameter and $\bar{I}$ is the input passed directly through the shortcut connection.

#### 5.4.2. Squeeze-And-Excitation Attention Mechanism

The SE attention mechanism enhances the representational capacity of the network by adaptively recalibrating channel-wise feature maps. This mechanism assigns greater importance to the most informative spectral channels while suppressing noise and irrelevant features [30]. By doing so, it ensures that the network effectively focuses on critical spectral information relevant to HSI classification, particularly for identifying AFB1 contamination in almonds. The SE mechanism operates in three key stages: squeeze, excitation, and recalibration, shown in Figure 5b.

The spatial dimensions of the input tensor U, with dimensions (H,W,D,C), are reduced through global pooling to produce a channel descriptor $z_{c}$ here c denotes the channel index. This operation summarizes the global information across all spatial dimensions:

(7) $$z_{c}=\frac{1}{HWD}\sum_{i,j,k}^{H,W,D}U_{i,j,k,c}$$

The channel descriptor $z_{c}$ is passed through two fully connected layers with learnable weights ($W_{1}$ and $W_{2}$) to compute the channel importance scores $s_{c}$. This process involves the ReLU activation function (δ) and the sigmoid function (σ) to produce normalized attention weights:

(8) $$s_{c}=\sigma (W_{2}\delta (W_{1}z_{c}))$$

The SE attention mechanism is implemented in the proposed AGIR-3DNet model through a ‘sigmoid’ layer that recalibrates channel-wise feature maps after the inception and residual blocks. This recalibration process emphasizes spectral features most relevant to AFB1 detection, improving the network’s focus on critical patterns within hyperspectral data. By integrating SE attention after feature extraction stages, the mechanism enhances the contributions of highly informative features from multi-scale convolutions and residual pathways, ensuring optimal utilization of the spectral-spatial information.

## Figures and Tables

**Table 1:** Performance comparison of classical machine learning and deep learning models for aflatoxin B1 classification.

Model Name	4 Spectra			6 Spectra			8 Spectra			Full Spectra		
	Acc	F1	AUC	Acc	F1	AUC	Acc	F1	AUC	Acc	F1	AUC
SVM [21]	76.40	0.75	0.76	77.38	0.75	0.77	78.13	0.76	0.78	76.54	0.75	0.77
QDA [21]	67.20	0.64	0.67	66.45	0.63	0.66	66.45	0.63	0.66	70.10	0.68	0.70
AdaBoost	70.05	0.68	0.70	70.33	0.68	0.70	71.36	0.68	0.71	75.05	0.73	0.75
RUSB	68.74	0.65	0.68	69.16	0.66	0.69	68.64	0.65	0.68	72.29	0.70	0.72
Subs	77.06	0.75	0.77	78.13	0.76	0.78	79.25	0.78	0.79	77.52	0.76	0.78
3D Inception [21]	82.70	0.83	0.89	85.44	0.84	0.93	86.92	0.86	0.94	78.70	0.75	0.87
3D Inception ResNet [21]	85.24	**0.86**	0.94	**89.41**	**0.89**	**0.96**	90.59	0.89	0.97	86.70	0.86	0.95
AGIR-3DNet	**87.03**	**0.86**	**0.95**	89.30	**0.89**	**0.96**	**93.30**	**0.94**	**0.98**	**88.53**	**0.88**	**0.97**

ACC, accuracy; F1, F1-score; AUC, area under the receiver operating characteristic curve; SVM, support vector machine; QDA, quadratic discriminant analysis; AdaBoost, adaptive boosting; RUSB, random under-sampling boosting; Subs, subspace ensemble learning; AGIR-3DNet, attention-guided inception-ResNet 3D network.

**Table 2:** Evaluation of the impact of parameter tuning on the proposed AGIR-3DNet.

Model Variant	Model Deepness	FLOPs (G)	Time (S)	Parameters (M)	Training			Testing			Validation		
					ACC	F1	AUC	ACC	F1	AUC	ACC	F1	AUC
AGIR-3DNet with SE	1× AGIR	44.7	**2.18**	10	96.78	0.97	0.99	91.78	0.92	0.97	91.14	0.90	0.97
	2× AGIR	88.6	2.34	17.8	98.39	0.98	0.99	93.92	0.93	0.98	93.30	0.94	0.98
	3× AGIR	132.6	2.36	25.6	96.22	0.94	0.96	90.01	0.90	0.94	89.12	0.89	0.94
AGIR-3DNet without SE	1× AGIR	40.27	2.41	6.9	91.65	0.91	0.97	88.42	0.88	0.96	87.27	0.88	0.95
	2× AGIR	79.84	2.43	13.1	93.72	0.94	0.98	89.11	0.88	0.96	90.60	0.91	0.97
	3× AGIR	119.4	3.75	19.4	96.86	0.97	0.99	91.89	0.92	0.92	92.32	0.91	0.98

ACC, accuracy; F1, F1-score; AUC, area under the receiver operating characteristic curve; G, giga; M, mega; SE, squeeze-excitation; AGIR, attention-guided inception–-ResNet. The testing time required for classifying 899 samples was 4.76 s for 3D Inception, 7.49 s for deep Inception-ResNet.

## Data Availability

Due to the data is our university’s intellectual property, the data presented in this study are available on request from the corresponding author.

## Supplementary Materials

The following supporting information can be downloaded at: https://www.mdpi.com/article/10.3390/toxins18020076/s1, Figure S1. HPLC chromatogram of the solvent blank (methanol–water mixture); Figure S2. HPLC chromatogram of almond sample contaminated with 250 ppb aflatoxin B1; Figure S3. HPLC chromatogram of almond sample contaminated with 500 ppb aflatoxin B1; Figure S4. HPLC chromatogram of almond sample contaminated with 750 ppb aflatoxin B1; Figure S5. HPLC chromatogram of almond sample contaminated with 1000 ppb aflatoxin B1.
