# Supplementary material for: Squeeze-Excitation Attention-Guided 3D Inception ResNet for Aflatoxin B1 Classification in Almonds Using Hyperspectral Imaging
Source: Toxins (Basel). 2026 Feb 2;18(2):76. doi: 10.3390/toxins18020076 (PMC12944700; doi:10.3390/toxins18020076)
Supplement: Supplementary file 1 [file toxins-18-00076-s001.zip › toxins-4081804-supplementary.pdf]

Supplementary Materials: Squeeze-Excitation Attention-Guided 3D Inception ResNet for Aflatoxin B1 Classification in Almonds Using Hyperspectral Imaging

Md. Ahasan Kabir, Ivan Lee and Sang-Heon Lee

The HPLC chromatograms (a few sample testing results) illustrate the separation and detection of aflatoxin B1 (AFB1) in almond samples as a function of retention time. A well-defined and reproducible AFB1 peak is consistently observed at around 9 minutes mark, confirming stable chromatographic conditions and accurate compound identification. The solvent blank (methanol–water) shows no detectable peaks at the AFB1 retention time, verifying the absence of background interference or carryover.

For contaminated almond samples, the AFB1 peak intensity and peak area increase progressively with rising contamination levels from 250 to 1000 ppb, demonstrating a strong concentration–response relationship. Image-based inspection of the chromatograms reveals clear peak shape symmetry and improved signal-to-noise ratios at higher concentrations, supporting reliable quantitative analysis. These chromatographic responses were used to construct calibration curves and calculate the actual AFB1 concentrations in almond samples, which served as reference ground-truth data for validating hyperspectral imaging–based detection models and ensuring analytical accuracy.

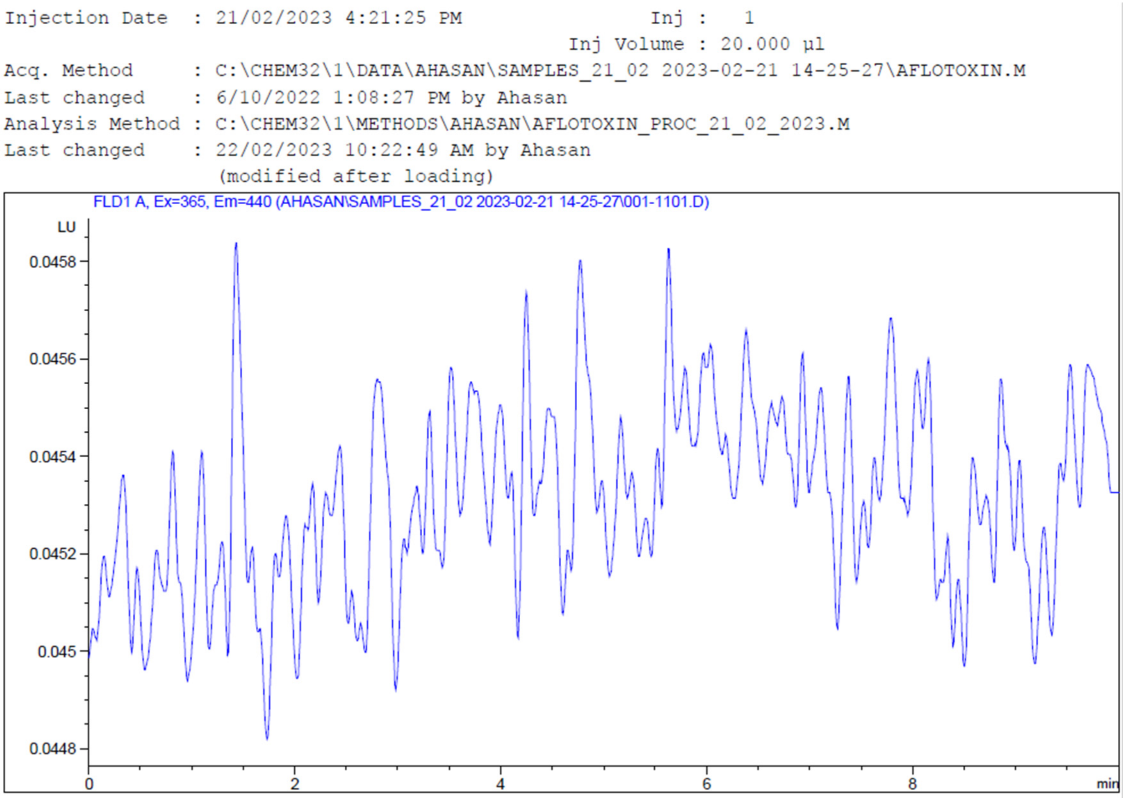

Figure S1. HPLC chromatogram of the solvent blank (methanol–water mixture).

```
Injection Date  : 21/02/2023 1:03:50 PM              Inj :   1
                                              Inj Volume : 20.000 µl
Acq. Method     : C:\CHEM32\1\DATA\AHASAN\CALIBRATION_21_02 2023-02-21 12-39-44\AFLOTOXIN.M
Last changed    : 6/10/2022 1:08:27 PM by Ahasan
Analysis Method : C:\CHEM32\1\METHODS\AHASAN\AFLOTOXIN_PROC_21_02_2023.M
Last changed    : 22/02/2023 10:22:49 AM by Ahasan
                  (modified after loading)
Additional Info : Peak(s) manually integrated
```

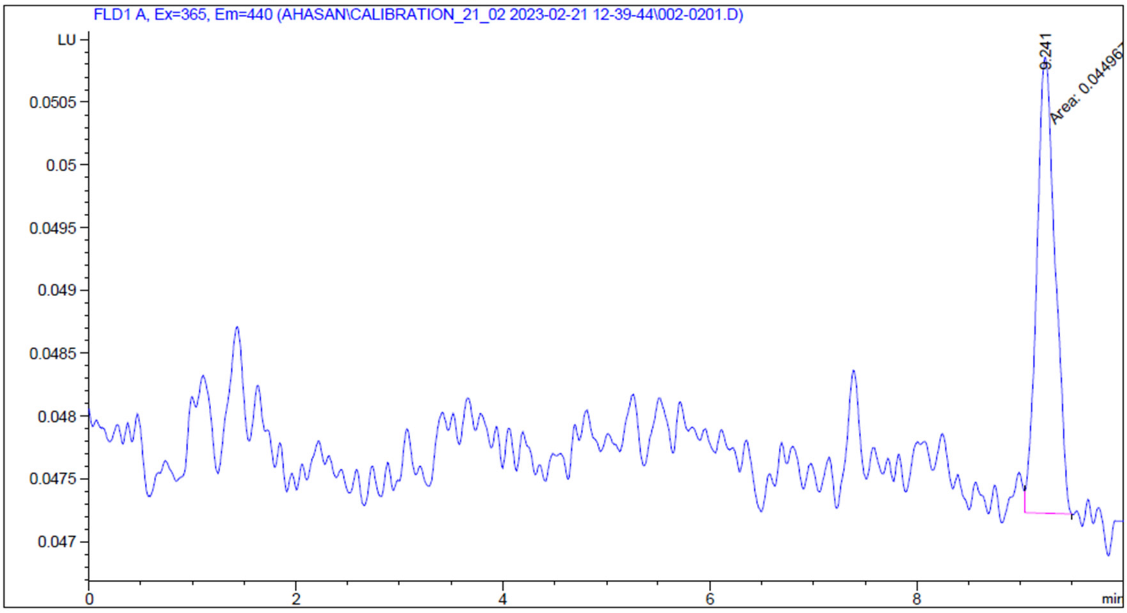

Figure S2. HPLC chromatogram of almond sample contaminated with 250 ppb aflatoxin B1.

```
Injection Date  : 21/02/2023 1:26:43 PM              Inj :   1
                                              Inj Volume : 20.000 µl
Acq. Method     : C:\CHEM32\1\DATA\AHASAN\CALIBRATION_21_02 2023-02-21 12-39-44\AFLOTOXIN.M
Last changed    : 6/10/2022 1:08:27 PM by Ahasan
Analysis Method : C:\CHEM32\1\METHODS\AHASAN\AFLOTOXIN_PROC_21_02_2023.M
Last changed    : 22/02/2023 10:22:49 AM by Ahasan
                  (modified after loading)
```

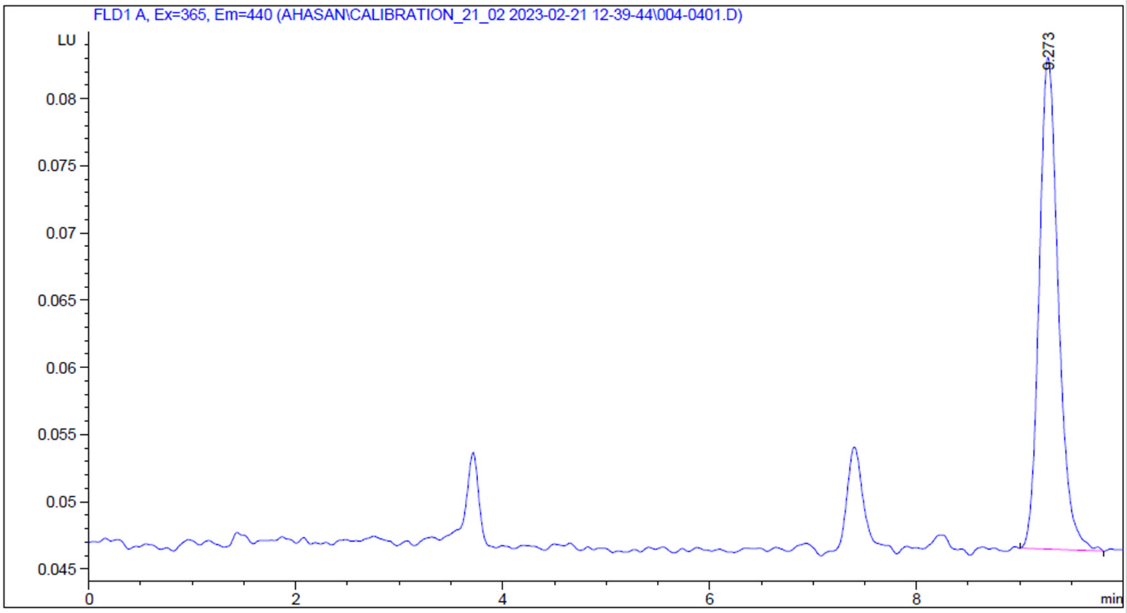

Figure S3. HPLC chromatogram of almond sample contaminated with 500 ppb aflatoxin B1.

```
Injection Date  : 21/02/2023 1:38:07 PM              Inj :   1
                                              Inj Volume : 20.000 µl
Acq. Method     : C:\CHEM32\1\DATA\AHASAN\CALIBRATION_21_02 2023-02-21 12-39-44\AFLOTOXIN.M
Last changed    : 6/10/2022 1:08:27 PM by Ahasan
Analysis Method : C:\CHEM32\1\METHODS\AHASAN\AFLOTOXIN_PROC_21_02_2023.M
Last changed    : 22/02/2023 10:22:49 AM by Ahasan
                  (modified after loading)
```

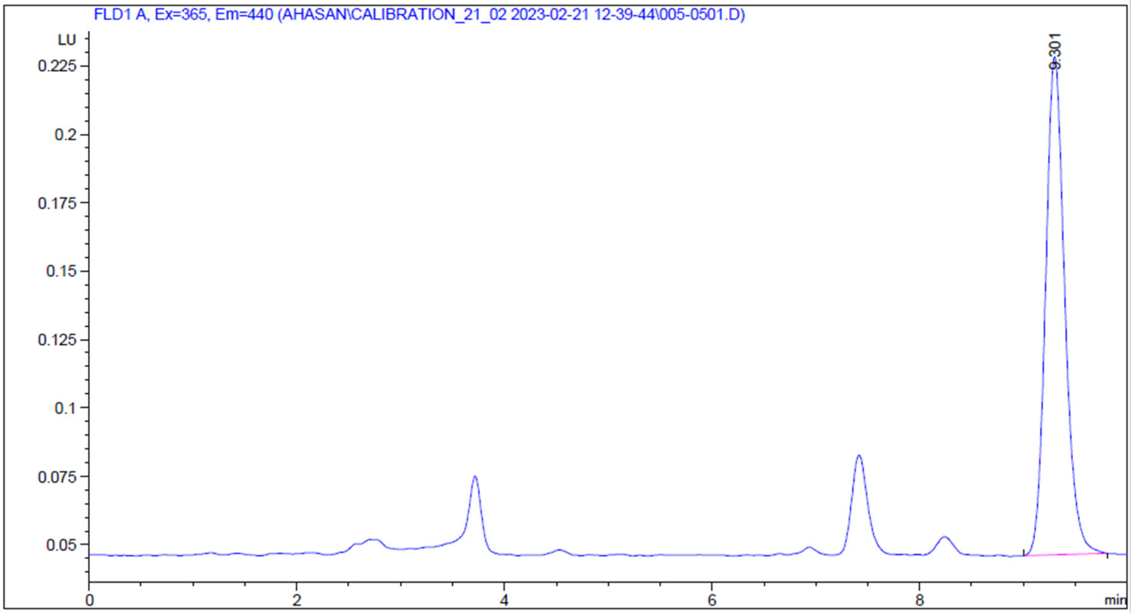

Figure S4. HPLC chromatogram of almond sample contaminated with 750 ppb aflatoxin B1.

```
Injection Date  : 21/02/2023 1:49:34 PM              Inj :   1
                                              Inj Volume : 20.000 µl
Acq. Method     : C:\CHEM32\1\DATA\AHASAN\CALIBRATION_21_02 2023-02-21 12-39-44\AFLOTOXIN.M
Last changed    : 6/10/2022 1:08:27 PM by Ahasan
Analysis Method : C:\CHEM32\1\METHODS\AHASAN\AFLOTOXIN_PROC_21_02_2023.M
Last changed    : 22/02/2023 10:22:49 AM by Ahasan
                  (modified after loading)
```

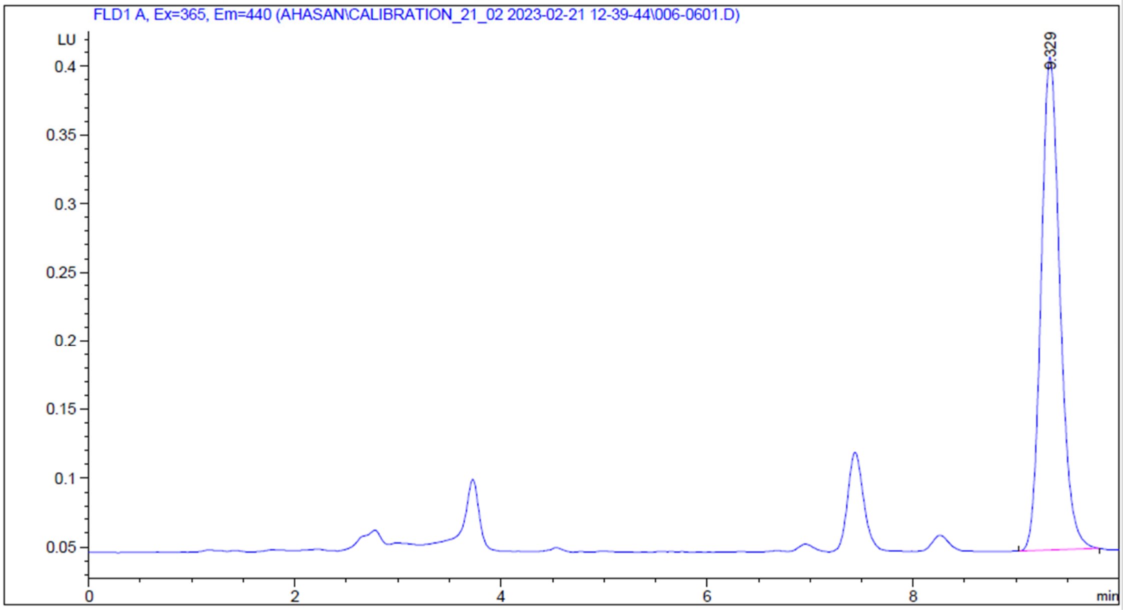

Figure S5. HPLC chromatogram of almond sample contaminated with 1000 ppb aflatoxin B1
